# Reactive Oxygen Species, Apoptosis, and Mitochondrial Dysfunction in Hearing Loss

**DOI:** 10.1155/2015/617207

**Published:** 2015-03-22

**Authors:** Teru Kamogashira, Chisato Fujimoto, Tatsuya Yamasoba

**Affiliations:** Department of Otolaryngology and Head and Neck Surgery, Faculty of Medicine, University of Tokyo, 7-3-1 Hongo, Bunkyo-ku, Tokyo 113-8665, Japan

## Abstract

Reactive oxygen species (ROS) production is involved in several apoptotic and necrotic cell death pathways in auditory tissues. These pathways are the major causes of most types of sensorineural hearing loss, including age-related hearing loss, hereditary hearing loss, ototoxic drug-induced hearing loss, and noise-induced hearing loss. ROS production can be triggered by dysfunctional mitochondrial oxidative phosphorylation and increases or decreases in ROS-related enzymes. Although apoptotic cell death pathways are mostly activated by ROS production, there are other pathways involved in hearing loss that do not depend on ROS production. Further studies of other pathways, such as endoplasmic reticulum stress and necrotic cell death, are required.

## 1. Introduction

Reactive oxygen species (ROS), such as hydroxyl radicals, superoxide anions, hydrogen peroxide, and singlet oxygen are mainly generated by the mitochondria in most mammalian cells [1, 2]. ROS, which are regarded as toxic products of cellular metabolism, can function as signaling molecules that regulate many physiological processes [3]. ROS play an important role in apoptosis induction under both physiological and pathological conditions; previous studies have shown that oxidative stress can cause cellular apoptosis via both the extrinsic cell death receptor pathway and the intrinsic mitochondrial cell death pathway [4]. The accumulation of ROS and subsequent apoptosis induction is an important contributor to several diseases and aging [5].

Elevated ROS formation and subsequent apoptosis induction have been implicated in the development of several hearing loss pathologies [6]. Furthermore, mitochondrial dysfunction plays an important role in some types of hearing loss [7]. In this review, we will focus on the involvement of ROS, mitochondrial dysfunction, and apoptosis induction in hearing loss pathology.

## 2. Cochlea and Hearing Loss

The cochlea is the auditory end organ of the inner ear. The organ of Corti is a core component of the cochlea and contains two types of sensory hair cells: inner and outer hair cells. When a sound pressure wave travels from the base to the apex of the cochlea, the basilar membrane of the cochlea vibrates [8]. The displacement of stereocilia—the mechanosensing organelles of the hair cells—results from basilar membrane vibration and opens the transduction ion channels. This causes potassium and calcium ions to enter, generating a transduction current that activates the voltage-dependent calcium channels along the lateral wall and base of the hair cell [9]. The inner hair cells release the neurotransmitter glutamate to encode acoustic signals for the postsynaptic afferent neuron [10]. The outer hair cells are much more sensitive to damage than the inner hair cells.

There are other components and supporting cells in the cochlea which maintain the structure and the environment of the cochlea. The stria vascularis and the spiral ligament are located at the lateral wall of the cochlea and generate the endocochlear potential (EP) [11]. EP is the positive voltage in the endolymphatic space of the cochlea and is essential for driving the potassium current through the transduction channels and stimulation of the hair cells [12]. The spiral ganglion is located at the modiolus and transfers the neuronal sound information from the hair cells to the brain. Several types of supporting cells are located in the cochlea and maintain homeostasis and the vibration of the cochlea; however, some of the mechanical functions are still under investigation [13].

Hearing loss is a reduction in sound sensitivity and is roughly divided into two types: acquired hearing loss and inherited hearing loss. The well-known types of acquired hearing loss are ototoxic drug-induced hearing loss (ODIHL), age-related hearing loss (ARHL), and noise-induced hearing loss (NIHL). The pathological features of each type of hearing loss are different. ODIHL is the commonly irreversible hearing loss caused by the intake of ototoxic drugs and the main mechanism is the loss of the hair cell [14]. NIHL is the partially irreversible hearing loss caused by exposure to loud sounds and the main mechanisms are mechanical damage to the organ of Corti and the loss of hair cells and the spiral ganglion [15]. ARHL, also known as presbycusis, is the progressive hearing loss associated with aging and the main mechanisms are the loss of hair cells, spiral ganglion cells, and stria vascularis cells [16]. The neural injury without hearing loss in noise exposure or aging is suggested in a mouse model [17]. Inherited hearing loss is caused by the dysfunction of some components of the cochlea, some of which are well researched, such as the hair cell [18], the tectorial membrane [19], and EP [20, 21].

## 3. Mitochondrial DNA Mutation Diseases Related to Hearing Loss

Many chromosomal genes encoding proteins have been associated with hereditary hearing loss, such as myosin [18], extracellular matrix [19], cadherin [22], ion channels [23], and transfer RNA (tRNA) or ribosomal RNA (rRNA) coding mitochondrial genes [24]. The mechanisms of mitochondrial dysfunction in certain gene mutations that encode mitochondrial rRNA or tRNA have recently been described. Hereditary hearing loss with aminoglycoside hypersusceptibility will be discussed in the next section.

Mitochondrial myopathy, encephalopathy, lactic acidosis, and stroke-like episodes (MELAS) syndrome [25] are all associated with hearing loss [26, 27]. MELAS mutations occur in mitochondrial genes [24, 26] and cause mitochondrial dysfunction. The mechanisms of mitochondrial translation are independent of chromosomal translation and the genes encoding mitochondrial tRNA are encoded in the mitochondrial DNA. The most common MELAS mutation is a 3243A>G mutation, which changes the structure of the mitochondrial leucine tRNA. Moreover, the third nucleotide of the anticodon loop of mitochondrial leucine tRNA is uracil and is modified by taurine modifying enzymes. The enzymes are hypothesized to be GTP-binding protein 3 (GTPBP3) and mitochondrial translation optimization 1 (MTO1) [28, 29] encoded on chromosomes. The structural change in tRNA inhibits the taurine modification of the uracil [30]. The modified anticodon loop of tRNA can pair adenine and guanine, the unmodified anticodon loop can pair adenine, and the unmodified tRNA inhibits the translation of UUG to leucine. The ND6 subunit of the mitochondrial oxidative phosphorylation (OXPHOS) enzyme complex I is encoded in mitochondrial DNA and its triplets hold the UUG codon; therefore, enzyme activity decreases in mitochondria with the 3243A>G mutation [31]. The decreased activity of the OXPHOS electron transport chain leads to an increased ROS production. This then induces the opening of nonspecific high conductance permeability transition pores in the mitochondrial inner membrane, decreased mitochondrial membrane potential, increased mitophagy, and apoptotic cell death [32]. MTO1 mutations show similar symptoms to MELAS [33], although their impact on the cochlear function is poorly understood.

The mutation rate of mitochondrial DNA differs between tissues. Rates are higher in the spiral ganglion cells and saccular macula than in the hair cells of the organ of Corti, the stria vascularis, and the facial nerve [34]. There is a good correlation between the mutation rate and histological findings [35]. These results indicate that the differences in OXPHOS activity between tissues affect both the organ activity and specific clinical symptoms.

## 4. Hearing Loss Induced by Ototoxic Drugs

Two types of ototoxic drug classes are widely known in clinical practice [36]: aminoglycoside antibiotics and platinum-based anticancer drugs. Both drug classes mainly damage the hair cells in the organ of Corti through ROS production via apoptotic pathways.

Aminoglycosides are broad-spectrum antibiotics that require a close monitoring of their potential ototoxicity and nephrotoxicity [37]. The nephrotoxicity is generally reversible because the cells of the proximal convoluted tubules of the kidney can proliferate and recover [38], but the ototoxicity is irreversible because the hair cells of the cochlea cannot proliferate and recover. Aminoglycosides probably damage the outer hair cells relative to the inner hair cells by triggering differential apoptotic signals [39]. In addition, the basal turn hair cells, which process high frequency sounds, are preferentially damaged compared with the hair cells in the apical turn, which process low frequency sound [40]. Thus, aminoglycoside use needs a careful clinical evaluation of the indication.

ROS are now established as the main initiators of aminoglycoside-induced hearing loss [41]. Aminoglycosides tend to accumulate in the mitochondria of the hair cells [42]; gentamicin directly inhibits protein synthesis in mitochondrial ribosomes [43] and triggers mitochondrial permeability transition pore opening [44].

The 1555A>G mitochondrial DNA mutation causes hereditary hearing loss with known aminoglycoside hypersusceptibility [45]. The 12S rRNA gene is encoded at the mutation and changes the rRNA conformation to bind with aminoglycosides more tightly than when normally configured [43]. However, the precise mechanism of aminoglycoside interaction with rRNA is still under investigation [46]. Other mutations of mitochondrial DNA associated with aminoglycoside hypersusceptible hearing loss have been found in a recent study [47].

Platinum-based anticancer drugs are frequently used in the treatment of many types of cancer including squamous cell carcinoma; adenocarcinoma; and undifferentiated carcinoma of the head, neck, lung, and bladder [48]. However, they have toxic effects on the cochlea, kidney, and neurons. The ototoxicity of cisplatin is widely known [49] and drugs that offer protection against ototoxicity have been studied [50]. Cisplatin acts as a DNA crosslinker in tumor cells, where its platinum atom binds to purine bases and inhibits cell proliferation, which inactivates the cell cycle and causes tumor cell apoptosis [51].

Cisplatin has both acute and chronic toxic effects on cochlear tissue. The acute effect is a reversible inhibition of transduction currents and voltage-dependent calcium currents in the hair cells [52] and the reaction of currents in the stria vascularis. The long lasting toxic reaction makes cochlear tissue to trigger ROS production and potassium conductance change [53], which cause apoptotic cell death [54]. These chronic effects are irreversible and the outer hair cells [55], the marginal cells of the stria vascularis [56], and the spiral ganglion cells [57] tend to degenerate when compared with the inner hair cells. The most common type of cisplatin ototoxicity results in a bilateral, high frequency, and sensorineural hearing loss [58].

ROS formations in the outer hair cells in response to cisplatin represent the binding of cisplatin to the sulfhydryl group of enzymes and the depletion of nicotinamide adenine dinucleotide phosphate (NADPH), copper, or selenium [59]. These processes are essential for glutathione peroxidase and glutathione reductase activity and NADPH oxidase activations [60]. NADPH oxidase 3 (NOX3), one of the six NADPH oxidases, is highly expressed in the organ of Corti [61]. Moreover, superoxide production of NOX3 increases under cisplatin treatment [61]. The other NADPH oxidases are also important in ROS production associated with cisplatin ototoxicity [60].

The increased ROS generation reduces the antioxidant defense mechanisms of the outer hair cells, causing the release of cytochrome c from mitochondria, activating the caspase pathways, and triggering apoptotic cell death [51]. The cytochrome c increase also activates caspases-3 and caspases-9, which trigger deoxyribonuclease activity [62].

Other potential apoptotic pathways in the stria vascularis of the lateral wall or in the spiral ganglion include the activation of nuclear factor kappa B (NF-*κ*B) and the formation of nitric oxide (NO) [56] and the activation of high-mobility group protein 1 (HMG1), NO production, and 4-hydroxynonenal (4-HNE) production [63]. Increased NO levels have been shown in a rat model [64] and increased NF-*κ*B and inductable nitric oxide synthase (iNOS) immunolabeling [65, 66] have been shown. These results indicate that NO and iNOS trigger apoptosis in the stria vascularis [66]. The higher level of HMG1 expression in modiolar tissue than kidney tissue [67] and the increase of iNOS in the spiral ganglion cells one day after cisplatin treatment [67] indicate that different apoptotic cell death pathways exist in the spiral ganglion.

## 5. Age-Related Hearing Loss

The prevalence of ARHL is expected to increase with aging population [7, 68–70]. Although many factors have been researched including environmental, hereditary, and medical factors [71, 72], the precise mechanism of ARHL is not yet understood.

The accumulation of mitochondrial DNA mutations is hypothesized to cause age-related degenerative diseases such as ARHL [73]. Increases of mitochondrial DNA mutations in cochlear tissue have been shown in humans [74]. Mitochondrial DNA replicates frequently and independently of the cell cycle and mitochondrial DNA mutations tend to accumulate more than chromosomal DNA mutations because mitochondrial DNA lacks protective histones. The same mechanism is suggested in mouse models of ARHL [75, 76]. The major mitochondrial DNA mutations occur in the genes encoding mitochondrial OXPHOS complexes and lead to dysfunctional OXPHOS activity. ROS formation in the dysfunctional mitochondria, decreased mitochondrial membrane potentials, and the activation of apoptotic pathways most likely causes hair cell death; however, other pathways are also hypothesized.

Because ROS play an important role in ARHL [77], the effects of supplementation of antioxidants against ARHL have been studied. In Fischer 344 rats, vitamin C, vitamin E, melatonin, and lazaroid had better effects in preserving auditory sensitivities and reducing mtDNA deletions than a placebo [78]. In C57BL/6 mice, vitamin C did not affect ARHL [79], but a combination of multiple antioxidant agents (L-cysteine-glutathione mixed disulfide, ribose-cysteine, NW-nitro-L-arginine methyl ester, vitamin B12, folate, and ascorbic acid) had significantly better effects on preserving auditory sensitivity than the control agents [80]. In CBA/J mice, supplementation with vitamin A, vitamin C, vitamin E, L-carnitine, and a-lipoic acid significantly increased the antioxidant capacity of inner ear tissues but did not improve the loss of hair cells and spiral ganglion cells and the progression of ARHL [81]. The prevention of ARHL by antioxidant supplementation is influenced by many factors, such as the type and dosage of antioxidant, the duration and timing of the treatment, and the species.

Intrinsic and extrinsic pathways are involved in apoptotic cell death in ARHL. The intrinsic pathway is mitochondrial dependent and is triggered by a loss of the mitochondrial membrane potential. The extrinsic pathway is triggered by ligands that bind to cell surface receptors [82, 83]. Furthermore, the prevention of ARHL following the deletion of the mitochondrial proapoptotic gene, brassinosteroid insensitive-1-associated receptor kinase (*Bak*) [84], indicates that the intrinsic apoptotic pathway is necessary for ARHL.

## 6. Noise-Induced Hearing Loss

Noise is also a major cause of hearing loss [85]. It is often associated with the military, clubs, discos, and portable audio players; the prevalence of noise-induced hearing loss is predicted to increase over the coming years [15].

Two main pathways result in cochlear damage following noise exposure: mechanical damage [86] and biochemical pathways triggering apoptosis or necrosis. The outer hair cells are much more sensitive to noise exposure than the inner hair cells. Morphological nuclear changes [87] and increases in apoptotic markers, such as caspase [88], tumor necrosis factor receptor [89], and associated promoters [90] occur in mouse or rat models. These indicate the importance of apoptotic pathways in noise-induced hearing loss.

Several pathways that trigger apoptotic cell death in noise-induced hearing loss have been studied in animal models. Studies have revealed common increases in ROS or similar reactive species [91] but ROS formation tends to decrease over time [92]. The mitochondrial release of apoptosis-inducing factor (AIF) and mitochondrial endonuclease G (EndoG) into the cytosol cochlear cells after noise exposure has been shown in guinea pig models [93]. The c-Jun N-terminal kinase (JNK) signaling mitogen-activated protein kinase (MAPK) pathways that mediate cells entering programmed apoptosis are also increased after sound trauma in guinea pig models [94]. In addition, JNK signaling pathways are activated by ROS formation [95] and other apoptotic pathways are hypothesized in noise-induced hearing loss.

Other pathways which do not depend on ROS production have been predicted. The increase of free Ca^2+^ in the outer hair cells [96] or the activation of Ca^2+^ and calmodulin-controlled calcineurin [97] may trigger apoptotic or necrotic cell death pathway without ROS production. The decrease in blood flow [98] caused by vasoactive products [99] leads to ischemia and may contribute to the damage of cochlear tissue. The excessive release of the neurotransmitter glutamate from the inner hair cells can trigger defects in the synaptic connections in the auditory nerve and cause spiral ganglion cell death [100].

## 7. Conclusion

ROS production and mitochondrial apoptotic pathways play important roles in many types of hearing loss. Major ROS production pathways include OXPHOS dysfunction, increased pro-ROS enzyme activity, and decreased anti-ROS activity. Hearing loss pathways vary and some remain under investigation. Other pathways, such as ER stress and necrotic cell death, are also involved in hearing loss. Further studies of each type of hearing loss are required including the investigation of ROS, apoptosis, and other types of cell death.
